# Amiodarone inhibits arrhythmias in hypertensive rats by improving myocardial biomechanical properties

**DOI:** 10.1038/s41598-020-78677-5

**Published:** 2020-12-10

**Authors:** Yifeng Nie, Yin He, Dong Han, Yuansheng Liu, Xiang Li

**Affiliations:** 1 Emergency Department, Peking University People’s Hospital, Beijing, 100044 People’s Republic of China; 2 CAS Center for Excellence in Nanoscience, National Center for Nanoscience and Technology, Beijing, 100190 People’s Republic of China; 3 School of Future Technology, University of Chinese Academy of Sciences, Beijing, 100049 People’s Republic of China; 4 Emergency Department, Beijing Anzhen Hospital, Capital Medical University, Beijing, 100029 People’s Republic of China

**Keywords:** Drug discovery, Cardiology, Diseases

## Abstract

The prevalence of arrhythmia in patients with hypertension has gradually attracted widespread attention. However, the relationship between hypertension and arrhythmia still lacks more attention. Herein, we explore the biomechanical mechanism of arrhythmia in hypertensive rats and the effect of amiodarone on biomechanical properties. We applied micro-mechanics and amiodarone to stimulate single ventricular myocytes to compare changes of mechanical parameters and the mechanism was investigated in biomechanics. Then we verified the expression changes of genes and long non-coding RNAs (lncRNAs) related to myocardial mechanics to explore the effect of amiodarone on biomechanical properties. The results found that the stiffness of ventricular myocytes and calcium ion levels in hypertensive rats were significantly increased and amiodarone could alleviate the intracellular calcium response and biomechanical stimulation. In addition, experiments showed spontaneously hypertensive rats were more likely to induce arrhythmia and preoperative amiodarone intervention significantly reduced the occurrence of arrhythmias. Meanwhile, high-throughput sequencing showed the genes and lncRNAs related to myocardial mechanics changed significantly in the spontaneously hypertensive rats that amiodarone was injected. These results strengthen the evidence that hypertension rats are prone to arrhythmia with abnormal myocardial biomechanical properties. Amiodarone effectively inhibit arrhythmia by improving the myocardial biomechanical properties and weakening the sensitivity of mechanical stretch stimulation.

## Introduction

Hypertension has the characteristics of high incidence rate, more complications, and prone to sudden death^1^, which has become one of the major diseases that mainly endanger the health of human. The incidence of arrhythmia such as atrial fibrillation and ventricular arrhythmia in patients with a long history of hypertension is obviously increased^2^. However, the relationship between hypertension and arrhythmia still lacks more attention. At present, studies have shown that left ventricular hypertrophy (LVH) occurs in hypertension patients, which is a compensatory mechanism for the heart to adapt to long-term post-load increase and is a marker of organic heart injury^3^. The occurrence of LVH is closely related to the increased incidence of ventricular arrhythmias and high mortality in hypertensive patients^4^. With the development of hypertension-related ventricular myocyte hypertrophy, it is more sensitive to mechanical stimulation, which plays a very important role in the occurrence of hypertension-related arrhythmias. Ventricular myocyte activity is closely related to mechanical stimulation, including excitation–contraction coupling and electromechanical feedback^5^, thus affecting the electrical signal process from mechanical stimulation to cardiac electrophysiological activity^6^. For example, mechanical stimulation can change the effective refractory period of ventricular myocytes action potentials, affecting Ca^2+^ transients in ventricular myocytes and increasing the susceptibility to ventricular arrhythmias^7^. In addition, stretch-activated ion channels (SACs) are a very important electromechanical feedback sensor that inhibits arrhythmias caused by stretch stimulation when SACs blockers are applied^8,9^. Therefore, hypertension promoting hypertrophy of ventricular myocytes and increasing sensitivity to mechanical stretch play an important role in the development of hypertension-related ventricular hypertrophic arrhythmia.

In recent years, the development and advancement of biomechanics have opened up a new vision in medical research. Atomic Force microscopy (AFM), as a powerful tool for studying biomechanics, has micromechanical receptors and micro-cantilevers, which could generate a tiny indentation force on the surface of myocardial cell membrane, thus resulting in micro-mechanical stretching and the data will ultimately be presented in the form of force–distance curve^10,11^. Therefore, this method can simulate the mechanical stimulation of hypertensive cardiac contraction on myocardial cells, thus making the mechanical research on myocardial cells more convincing. In this study, we first applied micro-mechanical stimulation to single ventricular myocytes in spontaneously hypertensive rats (SHR) and normal control rats (WKY) to compare the mechanical parameters of ventricular myocytes. In addition, laser confocal scanning microscope was used to observe the changes of intracellular Ca^2+^ concentration in ventricular myocytes by micro-mechanical stimulation, and the mechanism of hypertension and arrhythmia was investigated in biomechanics. However, amiodarone as an antiarrhythmic drug that blocks Na^+^, Ca^2+^, and K^+^ channels to treat atrial fibrillation and prevent sudden cardiac death^12^. There is no evidence for the electromechanical effect on ventricular myocytes in patients with hypertension and LVH. So we apply amiodarone to ventricular myocytes to observe the changes in the biomechanical properties, so as to study the effect of amiodarone on the mechanical properties of ventricular myocytes in hypertensive rats.

In recent years, it has been discovered that long non-coding RNAs (lncRNAs) those greater than 200 nt in length are widely expressed in eukaryotes and participate in various biological processes, which are closely related to human tumors, neurological disorders and cardiovascular diseases^13–16^. As key regulatory molecules and potential biomarkers of heart disease, lncRNAs are involved in regulating the occurrence and development of cardiac hypertrophy, arrhythmia, myocardial fibrosis and heart failure^17–20^. So we finally verified the expression changes of genes and long non-coding RNAs (lncRNAs) related to myocardial mechanics in hypertensive rats with arrhythmia by gene sequencing to explore the effect of amiodarone on biomechanical properties. This study is of great significance to the mechanism exploration, prevention and treatment of hypertension and arrhythmia diseases, and also provides a new idea for the research and development of new antiarrhythmic drugs.

## Results

### Stiffness and force curve changes of SHR and WKY ventricular myocytes at different indentation depths

**Figure 1:**
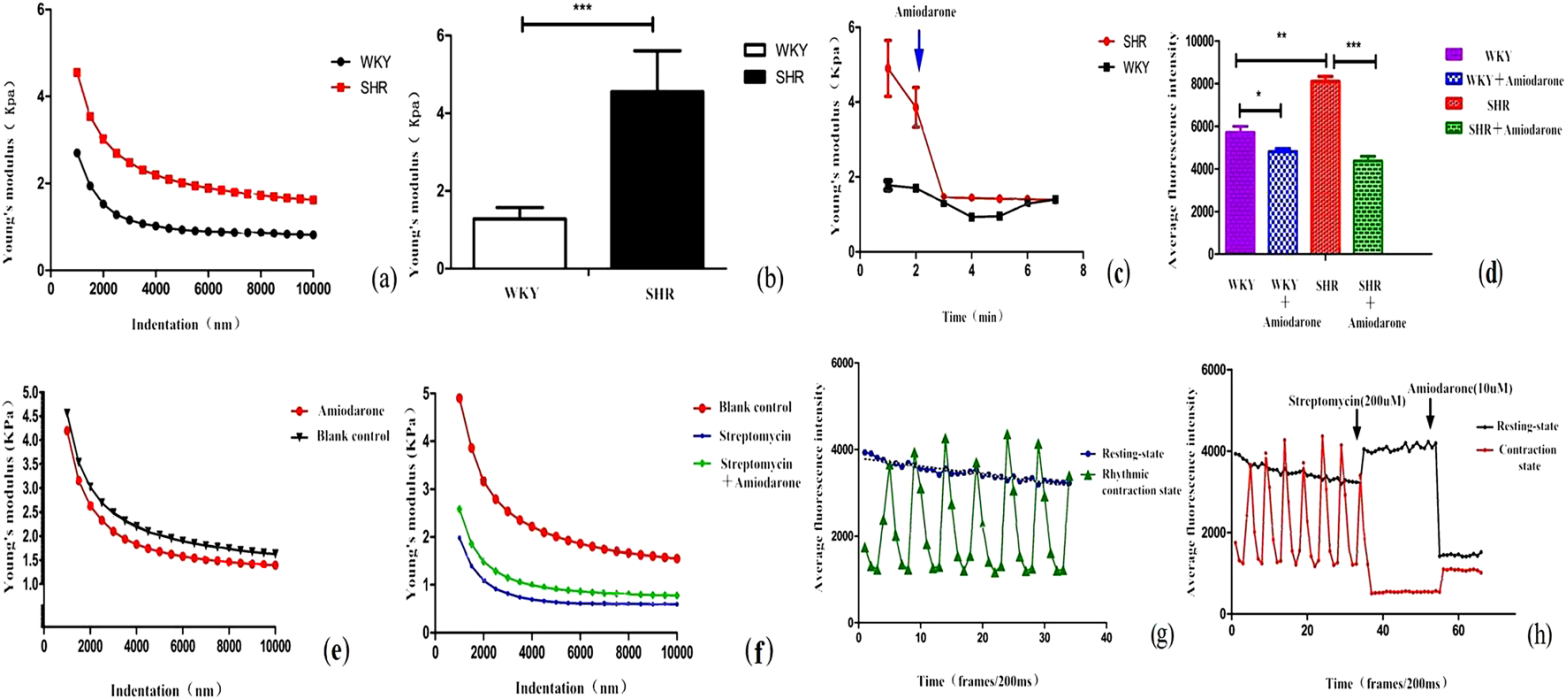
The effect and mechanism of amiodarone on ventricular myocytes in vitro. (**a**) Stiffness variation trend of ventricular myocytes in SHR and WKY at different indentation depths. (**b**) Young's modulus of SHR ventricular myocytes vs. Young's modulus of WKY ventricular myocytes. (**c**) Comparision of amiodarone's effect on the stiffness of ventricular myocytes in SHR group and WKY group: amiodarone was given within the second minute, Young's modulus of both groups decreased, but SHR decreased more significantly. (**d**) Comparison of amiodarone's effect on the stiffness of ventricular myocytes in SHR group and WKY group: amiodarone was given within the second minute, the average fluorescence intensity of both groups decreased, but the average fluorescence intensity of SHR group decreased more significantly. (**e**) Effect of amiodarone on stiffness of ventricular myocytes: comparison of stiffness before and after amiodarone added. (**f**) Effect of amiodarone on ventricular myocyte stiffness under streptomycin blocked. (**g**) Changes of intracellular Ca^2+^ concentration in ventricular myocytes in resting state and rhythmic contracting state. (**h**) The effect and mechanism of amiodarone on intracellular Ca^2+^ concentration in ventricular myocytes in resting state and rhythmic contracting state.

The stiffness changes at different indentation depths of ventricular muscle are shown in Fig. 1. As shown in Fig. 1a, the stiffness of SHR and WKY ventricular myocytes gradually decreased at different indentation depths, and the stiffness of the cells remained unchanged until the indentation depth of 2500–4000 nm. In addition, the Young's modulus has a significant difference in SHR group and WKY group and the stiffness of ventricular myocytes in the SHR group was significantly higher than that in the WKY group (4.87 ± 1.08 kPa vs. 1.28 ± 0.87 kPa, P = 0.00055 < 0.001, as shown in Fig. 1b).

### Mechanism of amiodarone on stiffness of ventricular myocytes

Young's modulus of ventricular myocytes in SHR group and WKY group decreased under the intervention of amiodarone (10 μmol/L) in the second minute, but the stiffness of ventricular myocytes in SHR group decreased more significantly (Fig. 1c). The average fluorescence intensity of ventricular myocytes in SHR group and WKY group decreased under the intervention of amiodarone, but the decrease was more obvious in SHR group (Fig. 1d). Streptomycin was used to block stretch-activated non-selective cation channels and then amiodarone was given to verify again to investigate whether amiodarone acted on the stiffness of ventricular myocytes under rhythmic contracting through stretch-activated non-selective cation channels. After streptomycin was given to block, the stiffness of ventricular myocytes was significantly reduced. While amiodarone was given again, stiffness was increasing (Fig. 1f).

### Effects of amiodarone on changes of Ca^2+^ in intraventricular myocytes

The intracellular Ca^2+^ concentration and average fluorescence intensity in ventricular myocytes of SHR group was higher than that of WKY group (Fig. 1d).

There were significantly different of Ca^2+^ concentration in intraventricular myocytes between ventricular myocytes in resting state and rhythmic contracting state (Fig. 1g).In addition, amiodarone had different effects on intracellular Ca^2+^ concentration in ventricular myocytes in different conditions (Fig. 1h).In resting state, streptomycin was given to block, Ca^2+^ concentration in intraventricular Myocytes increased and then amiodarone was given again, intracellular Ca^2+^ concentration in ventricular myocytes decreased significantly.

### Acute arrhythmia induced by rapid ascending hypertension through coarctation of aortic arch

**Figure 2:**
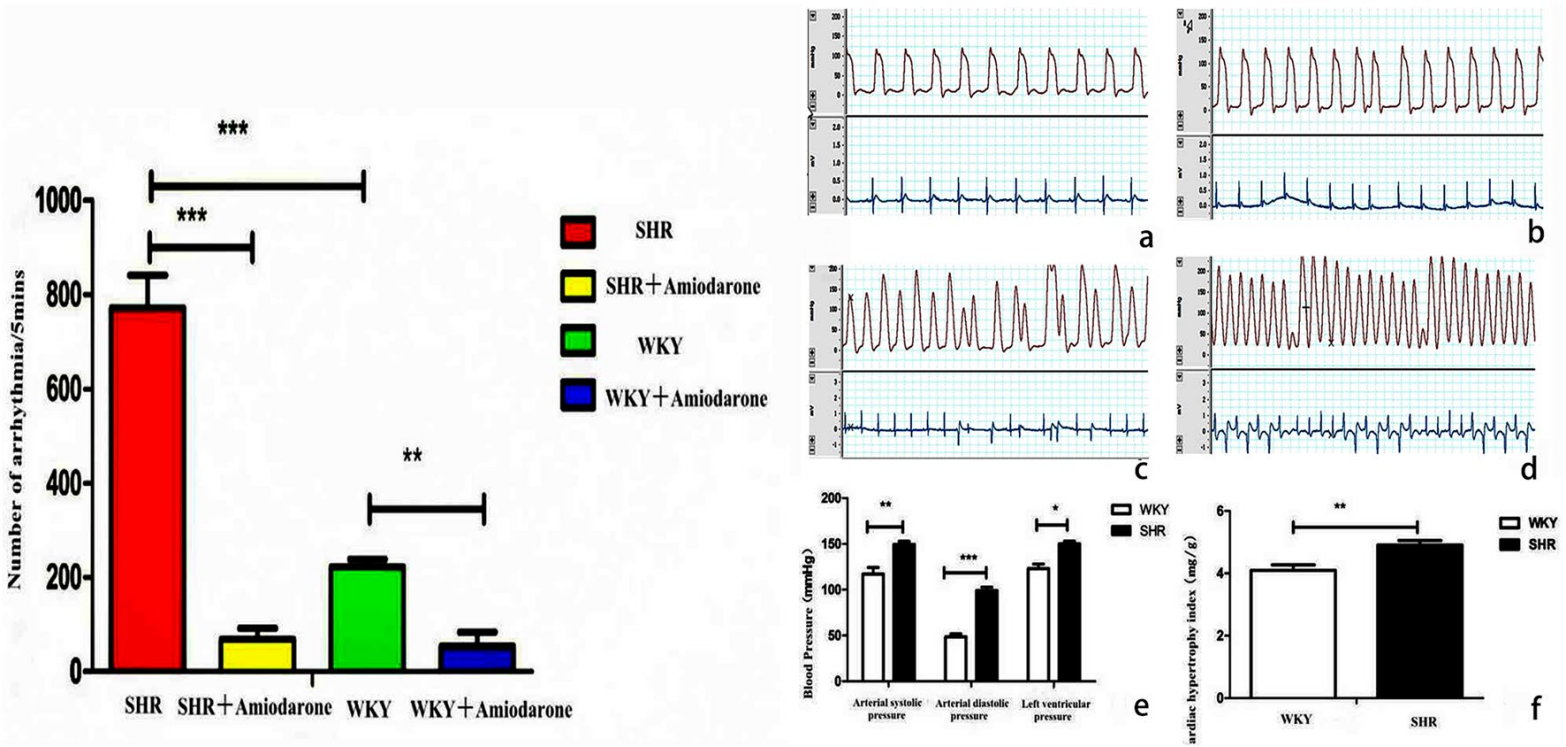
The effect of amiodarone on the animals in vivo. (**a**) Left ventricular pressure and electrocardiogram before aortic ligation in WKY group. (**b**) Left ventricular pressure and electrocardiogram before aortic ligation in SHR group. (**c**) Left ventricular pressure and electrocardiogram after aortic ligation in WKY group. (**d**) Left ventricular pressure and electrocardiogram after aortic ligation in SHR group. (**e**) Comparison of arterial pressure and left ventricular pressure between SHR and WKY rats. (**f**) Comparison of cardiac hypertrophy index between SHR and WKY rats.The total number of acute arrhythmias occurred within 5 min after coarctation of aortic arch for rapidly ascending hypertension (*representing P < 0.05, * * stands for P < 0.01, * * * represents P < 0.001).

After coarctation of aortic arch, the blood pressure and heart rate increased and various arrhythmias occurred quickly in SHR group and WKY group rats, which ventricular arrhythmias were the most common and followed by supraventricular arrhythmias (Fig. 2).Statistics showed that the total number of arrhythmias occurred within 5 min through coarctation of aortic arch was significantly higher in SHR group than that in WKY group. When amiodarone hydrochloride tablets were given, the number of arrhythmia in SHR group and WKY group within 5 min during the operation was significantly reduced and the SHR group was more obvious (SHR group vs. SHR Amiodarone group, 772 ± 154.66 times/5 min vs. 69 ± 52.59 times/5 min, P = 0.0063 < 0.001. WKY group vs. WKY Amiodarone group, 222.8 ± 36.46 times/5 min vs. 54 ± 47.37 times/5 min, P = 0.039 < 0.05, as shown in Fig. 2). The average arrhythmia reduction rate of amiodarone within 5 min was 91.06% vs. 75.76% in SHR group vs. WKY group.

### Comparison of arterial pressure, left ventricular pressure and cardiac hypertrophy index between SHR and WKY rats

The arterial pressure and the left ventricular pressure of rats were measured by carotid artery intubation. By comparing the measurement results of two groups, it was found that systolic pressure, diastolic pressure and left ventricular pressure of SHR rats were higher than those of WKY group (Fig. 2e). After measuring the heart weight and body weight of rats, we finally obtained the cardiac hypertrophy index of rats (cardiac hypertrophy index = heart weight/body weight, mg/g)^21^. The comparison between the two groups found that the cardiac hypertrophy index of SHR group rats was significantly higher than that of WKY group (Fig. 2f), illustrating that the 18-week-old SHR group of male rats not only had higher peripheral arterial pressure, but also had higher left ventricular pressure than WKY group rats, and the cardiac hypertrophy also occurred in rats.

### Effects of amiodarone on lncRNAs expression in SHR and WKY rats with arrhythmia

**Figure 3:**
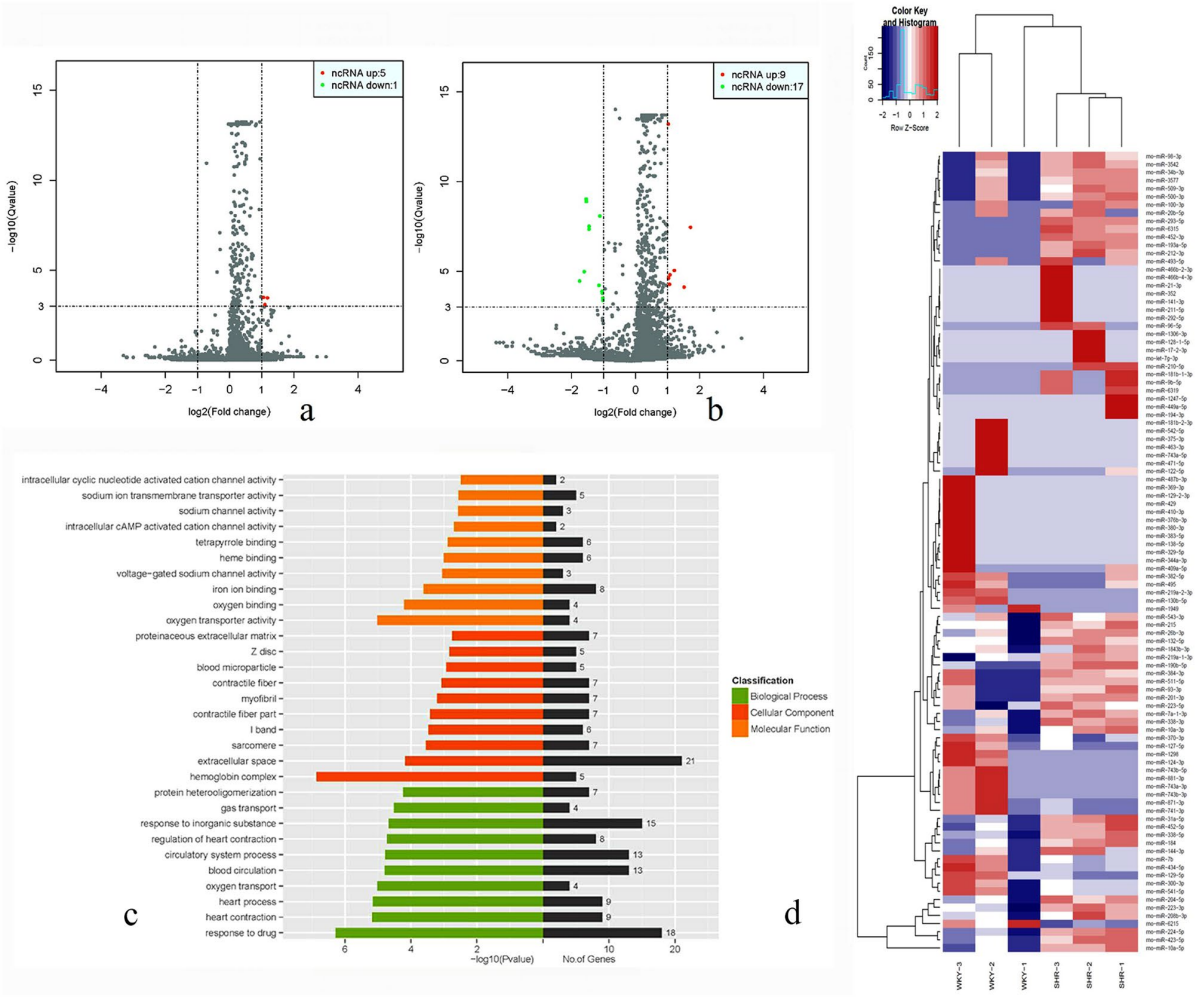
Effects of amiodarone on genes and lncRNAs expression. (**a**) Volcano plots of SHR group in arrhythmia treated with amiodarone. (**b**) Volcano plots of WKY group with arrhythmia treated with amiodarone. (**c**) GO analysis diagram for comparison of lncRNAs before and after amiodarone treatment in SHR group, green parts indicated biological processes and the red represented cellular components, and the yellow represented molecular functions. In addition, the black represented the number of related genes. (**d**) The cluster diagram of differential gene expression patterns. Column and row respectively represents experimental samples and the degree of gene expression in different samples: red means a relatively high expression and navy blue means low expression level. The sample tree on the top represents the similarity clustering relationship among the samples and the gene tree on the left represents the expression-similarity clustering relationship between genes^22^.

After amiodarone used, five kinds of lncRNAs were up-regulated and only a kind of lncRNAs were down-regulated in SHR group (Fig. 3a and Table S4 in Supplementary Material). In WKY group, nine kinds of lncRNAs expressions were up-regulated and seventeen kinds of lncRNAs expressions were down-regulated (Fig. 3b and Table S3 in Supplementary Material). Please refer to GO analysis (as shown in Fig. 3 and Fig. S3 in Supplementary Material) and genes with obvious differential expression related myocardial mechanics between the two groups of rats were obtained (Table.1).

**Table 1:** Expression differences of genes related to cardiac mechanics in SHR group after amiodarone treatment and 12 biological processes related to cardiac mechanics.

Biological processes related to cardiac mechanics	Number	P value	Name of participating gene
Positive regulation of striated muscle contraction	2	0.007	Ptgs2;Hsp90aa1
Positive regulation of vasoconstriction	2	0.008	Fgg;Ptgs2
Vascular morphology	5	0.022	C3;Ntrk2;Ptgs2;Hmox1;C6
Positive Regulation of Smooth Muscle Cell Proliferation	2	0.023	Ptgs2;Hmox1
Regulation of vasoconstriction	2	0.026	Fgg;Ptgs2
Blood pressure regulation	3	0.027	Ptgs2;Hmox1;Nrg1
Positive regulation of myocardial contraction	1	0.028	Hsp90aa1
Vascular development	5	0.038	C3;Ntrk2;Ptgs2;Hmox1;C6
Positive regulation of circulatory system	2	0.044	Fgg;Ptgs2
Intracellular calcium signaling	1	0.044	Ntrk2
Circulating system regulation	3	0.045	Fgg;Ptgs2;Hsp90aa1
Vasoconstriction	2	0.046	Fgg;Ptgs2

## Discussion

Herein, the biomechanical mechanism of arrhythmia in hypertensive rats and the effect of amiodarone on biomechanical properties were explored. As the indentation depth continued to increase, the ventricular myocytes in the SHR group were more sensitive to the stress response than the WKY group, and the changes were more severe (Fig. 1a).The micromechanical properties of ventricular myocytes in SHR group are abnormal and are more sensitive to mechanical stretch stimulation and more violent in response, which is related to the abnormal changes of Ca^2+^ in ventricular myocytes. The stiffness of ventricular myocytes decreased with the depth of indentation, when the depth of indentation was 2500–4000 nm to maintain a stable level at 3.47 ± 0.78 kPa and the stiffness of ventricular myocytes was obviously reduced and maintained at 2.08 ± 0.66 KPa with amiodarone added to the alleviate biomechanical stimulation(Fig. 1e). In additon, the stiffness of ventricular myocytes and Ca^2+^ levels in hypertensive rats were significantly increased than that in normal controls, so amiodarone could alleviate the intracellular calcium response and biomechanical stimulation.

Then the Ca^2+^ concentration in ventricular myocytes tended to be stable in the resting state, but in the rhythmic contracting state, the concentration of Ca^2+^ fluctuated regularly up and down around a certain axis with the change of time, which was called “calcium wave”^23,24^. Streptomycin could increase intracellular Ca^2+^ concentration in ventricular myocytes in resting state that was opposite to the effect of streptomycin on intracellular Ca^2+^ concentration in ventricular myocytes with previous micro-regional mechanical stimulation, which indicated that streptomycin could not block stretch-activated non-selective cation channels in resting state. However, in the state of rhythmic contraction of ventricular muscle, the intracellular Ca^2+^ concentration decreased significantly with streptomycin blocking and the calcium wave activity was inhibited. When amiodarone was given again, the intracellular Ca^2+^ concentration of ventricular muscle cells increased (Fig. 1f), which showed streptomycin could block stretch-activated non-selective cation channels under the rhythmic contracting condition and amiodarone did not work through acting on stretch-activated non-selective cation channels, instead, amiodarone works micro-regional mechanical stimulation to regulate Ca^2+^ levels.

The traditional methods of arrhythmia research are mostly from inflammation, ion channels, electrophysiological reconstruction and other aspects^25–28^, which could be related to cellular stress response. Previous evidences have shown that there is close connection between cardiac electrical activity and mechanical stimulation, including excitation–contraction coupling and electromechanical feedback. The electrical activity of cardiac myocytes in hypertension is closely related to the increased mechanical stimulation of cardiac afterload, so we investigate relevant mechanism from the biomechanical response of arrhythmia in hypertensive rats and the effect of amiodarone on biomechanical properties. Amiodarone is a common clinical antiarrhythmic drug that can block Ca^2+^ channels. However, there is no evidence for the biomechanical effect on ventricular myocytes in patients with hypertension and LVH. The effect of amiodarone on ventricular myocytes and animals was applied to observe the changes in the biomechanical properties, we found that amiodarone inhibited arrhythmias in hypertensive rats by improving myocardial biomechanical properties and weakened the sensitivity of mechanical stretch stimulation, which may be a potential direction to use drugs with mechanics-related targets for the trial of arrhythmia in the future.

In addition, there are significant differences in cardiac mechanics-related genes and LncRNAs between before and after amiodarone administration in hypertensive rats though transcriptome sequencing and twelve biological processes related to cardiac mechanics are discovered (Fig. 3 and Table 1) and 30 genes and 32 lncRNAs were identified to be differentially expressed (≥ twofold change), indicating that the expression of genes and lncRNAs are upregulated or downregulated in SHR group and WKY group. GO and KEGG pathway analysis were applied to explore the potential genes and lncRNAs functions, 12 biological processes related to cardiac mechanics including vascular development and vasoconstriction and blood pressure regulation and so on, further illustrating that amiodarone inhibits arrhythmias in hypertensive rats by improving myocardial biomechanical properties. These results shed some light on lncRNAs’ physiologic functions and provide useful information for exploring potential therapeutic treatments for arrhythmia.

## Conclusions

Arrhythmia caused by hypertension is closely related to the abnormal biomechanical characteristics and the increased sensitivity of heart to mechanical stretch stimulation, which is one of the biomechanical mechanisms of hypertension prone to arrhythmia. Amiodarone effectively inhibit arrhythmia by improving the myocardial biomechanical properties and weakening the sensitivity of mechanical stretch stimulation, which should be one of the biomechanical mechanisms of amiodarone in antiarrhythmic. This study is of great research significance for the mechanism exploration, prevention and treatment of hypertension and arrhythmia diseases and also provides a new idea for the research and development of new antiarrhythmic drugs.

## Materials and methods

### Experimental animals

The first research was divided into Spontaneously Hypertensive Rat(SHR) group (N = 20) and Wistar-Kyoto Rat (WKY) group (N = 20) in which set 18 weeks of male SHR as experimental objects, and WKY for experimental comparison to obtain a single myocardial cell by acute myocardial separation technology. And then each group were respectively and randomly assigned into blank group (N = 10) and amiodarone group (N = 10) again. The second part was grouped by SHR group (N = 10), WKY group (N = 10), SHR with arrhythmia group (N = 10), WKY with arrhythmia group (N = 10). Each group was respectively divided into control group (N = 5) and amiodarone group (N = 5) again. These experiments had been approved by the Ethics Committee of Peking University People's Hospital and all procedures were performed in accordance to the relevant guidelines and regulations (The detailed descriptions of reagents and equipment are in the Supplementary Material).

### Isolation and fixation the ventricular myocytes of adult rats

First, adult rats were intraperitoneally injected with chloral hydrate (3.5%) and sublingually injected with heparin sodium (0.3%). Then opened the chest and pericardium, exteriorized the heart, and cut the left and right ventricles into small pieces of about 1.0 mm^3^ to 4 °C normal saline, and then digested with collagenase type II solution, adding 1% of bovine serum albumin. After being blown, centrifuged, resuspended, bathed, etc. and finally obtained adherent ventricular myocytes (The detailed methods is in the Supplementary Material).

### Staining and dynamic monitoring of calcium ion in ventricular myocytes

Briefly, 3 μl calcium ion fluorescent probe Fluo-4/AM storage solution and 97 μl of low calcium KH solution mixed and add to the adherent ventricular myocytes, and then added 0.2 mol/L low calcium KH buffer at 37 °C for 30 min in the dark to complete the staining of calcium ions in ventricular myocytes. Subsequently, using the atomic force microscope (AFM) to monitor the calcium ion concentration dynamically.

### Collection cell force curve of ventricular myocytes

The micro-cantilever modified by glass microspheres (CSG11, elastic coefficient κ = 0.069 N/m, force sensitivity coefficient s = 325.96 nm/V, glass microspheres d ≈ 18 μm) was installed on the scanning head of atomic force microscope Bio-AC system. Then adjusted the laser position and selected contact mode to collect the force curve of ventricular myocytes in three different parts, and the collection was repeated for 3 times at intervals of more than 5 s for each part.

### Observation amiodarone's effect on intracellular calcium concentration in ventricular myocytes and collection the force curve

Ten force curves were collected from different parts of the myocardial cells by the above methods, and amiodarone (10 μmol/L) was added after the second minute, and a total of ten force curves were collected again after waiting for the static lag of liquid level.

### High-throughput sequencing of genes and lncRNAs in atrial cells and ventricular myocytes

The left ventricular tissues of three rats in each group were randomly selected for high-throughput sequencing of genes and lncRNAs^29,30^. After extracting total RNA from Trizol (Invitrogen, USA), rRNA was fragmented by kit, reverse-transcribed into single-stranded cDNA and purified^31–33^. Subsequently, the end-repair and the linker primer were added with PCR amplification and purification^34–36^. Then the library was qualitatively examined by Agilent 2200 TapeStation, and finally sequenced on the machine. The lncRNAs discussed in this article are all from authoritative databases such as RefSeq, UCSC Knowngenes, Ensembl and other related databases^37,38^ (The detailed methods is in the Supplementary Material).

### Statistical analysis

Statistical analysis was performed with SPSS 21.0 software. Student's t-test applied for comparison between two groups and the P value was corrected by the False Discovery Rate (FDR) and P < 0.05 was considered to be statistically significant.

## Supplementary Information

Supplementary Information

## Data Availability

The datasets generated during or analysed during the current study are available from the corresponding authors on reasonable request.
