# Supplementary material for: Amiodarone inhibits arrhythmias in hypertensive rats by improving myocardial biomechanical properties
Source: Sci Rep. 2020 Dec 10;10:21656. doi: 10.1038/s41598-020-78677-5 (PMC7730129; doi:10.1038/s41598-020-78677-5)
Supplement: Supplementary file 1 — Supplementary Information [file 41598_2020_78677_MOESM1_ESM.docx]

**Supplementary Information**

**Amiodarone Inhibits Arrhythmias in Hypertensive Rats by Improving Myocardial Biomechanical Properties**

*Yifeng Nie^1,2,3^, Yin He^1,4^, Dong Han^2,3^, Yuansheng Liu^1^*, and Xiang Li^2,3^**

^1^Emergency Department, Peking University People’s Hospital, Beijing,

100044, People’s Republic of China

^2^CAS Center for Excellence in Nanoscience, National Center for Nanoscience and Technology, Beijing, 100190, P.R China. ^3^School of Future Technology, University of Chinese Academy of Sciences, Beijing 100049, P.R China.

^4^Emergency Department, Beijing Anzhen Hospital, Capital Medical University, Beijing, 100029, P.R China.

*Corresponding Author : Yuansheng Liu

Emergency Department,

Peking University People’s Hospital,

Beijing, 100044, People’s Republic of China

E-mail address: lyspku@126.com

*Corresponding Author : Xiang Li

CAS Center for Excellence in Nanoscience,

National Center for Nanoscience and Technology,

Beijing, 100190, P.R China

E-mail address: lixiang@nanoctr.cn

**Experimental Section**

**Main Experimental Materials and Reagents**

Materials in the experiments included collagenase type II (309 Umg-1, Worthington, USA), Amiodarone hydrochloride injection (10 umol/L, Sanofi, France), bovine serum albumin IV (Sigma, US), Laminin (Roche, USA), Fluo-4, AM (5 mmol L-1, 1.5 μL, Biotium USA), Taurine (Bio Basic Inc, Canada), Creatine (Bio Basic Inc, Canada), HEPES (Sigma, USA), Dimethyl sulfoxide/DSMO (Sigma, USA), the oxygenated KH buffer was used to prepare the following solutions: collagenase II (160 U/ml), 1% BSA (1% BSA, 5 mmol/L taurine, 5 mmol/L creatine), Low calcium solution (1 mmol/L CaCl_2_, 1% BSA), high calcium solution (0.1 mol/L CaCl_2_). Other solutions were prepared with deionized water.

Reagents required for gene sequencing: TRIzol reagent (Life Technologies, USA), RNAScreenTape and RNA Reagent (Agilent Technologies, USA), D1000 ScreenTape and D1000 Reagent (Agilent Technologies, USA), EpicentreRibo-Zero rRNA Removal Kit (Illumina, USA), NEBNext® Ultra^TM^ RNA Library Prep Kit for Illumina (NEB, USA), Qubit® dsDNA HS Assay Kit (Life Technologies, USA), HiSeq Rapid SBS Kit V2 (200 cycle) (Illumina, USA), HiSeq Rapid PE Cluster Kit V2 (Illumina, USA), HiSeq 3000/4000 SBS Kit (300 Cycles) (Illumina, USA), HiSeq 3000/4000 PE Cluster Kit (Illumina USA).

**Main Experimental Instruments**

Atomic Force Microscope (Model 5500, Agilent Technologies, USA), High Speed Laser Confocal Scanning Microscope(A1, Nikon, Japan), Centrifuge (Primo R, Heraeus, Germany), Ultrapure Water Machine (Milli-Q Biocel Millipore, USA), Water bath shaker (SHY-2A, Jintan Experimental Instrument Factory, China), Constant Flow Pump (LEAD-2, Lange, China) and Agilent 2200 TapeStation (Agilent Technologies, USA),ND-1000 Nanodrop (Thermo Fisher, USA), Qubit 2.0 (Life Technologies, USA), Hiseq 3000/Hiseq 2500 (Illumina, USA).

**Establishment of Arrhythmia Model in Spontaneously Hypertensive Rats**

First, systolic blood pressure of the living rat was measured 10 times at a fixed time by the caudal arterial pressure and the heart rate was measured. Then the right common carotid artery of anesthetized rats was isolated and the distal end of heart was ligated. A plastic catheter filled with heparin physiological saline (with an inner diameter of 1 mm) was inserted into the artery and ligated, and the biological signal recording and analysis system was connected via a pressure transducer for measuring blood pressure. Left ventricular pressure (LVP), peripheral arterial systolic pressure, and peripheral vasodilatation pressure were measured though the catheter in the common carotid artery inserting into the left ventricle along the direction of the aorta. Finally the limb lead electrocardiogram was measured to record and analyze the duration and malignancy of arrhythmias in rats.

**Isolation and Fixation of the Ventricular Myocytes in Adult Rats**

Adult rats were anesthetized by intraperitoneal injection of chloral hydrate (3.5%) and anticoagulated by sublingual intravenous injection of heparin sodium (0.3%). Opened the thoracic cavity and pericardium, took out the heart together with the aortic root, placed it

in 4ºC normal saline. The heart was quickly suspended on a self-made Langendorff which was a device with constant pressure (perfusion pressure was 70 cm H2O). The heart was perfused with calcium-free K-H solution at 37 ºC for 3 minutes, then the heart was perfused with collagenase type II solution 80 ml for 20 minutes until the heart became soft, swollen and white, and the perfusion was stopped. Cut off the left and right ventricles and placed them in a plate, cut them into small pieces of about 1.0 mm3, add 15 ml of collagenase type II solution, and placed them in a 37 ºC shaking water bath to continue digestion (100 rpm, 10 minutes). Then took out the digested cell suspension, added 1% bovine serum albumin, resuspend cells. The supernatant was removed, 1% bovine serum albumin was added again, and the cells were resuspended for 15 minutes. Removed the supernatant, and finally added 1% bovine serum albumin 5 ml to resuspend the cells and added 0.1 mol/L CaCl2 solution 10 μl, 25 μl, 50 μl, mixed and placed in 37ºC water bath (100 rpm, 3 minutes). The ventricular myocytes after calcium supplementation were placed at room temperature.

**Staining and Dynamic Monitoring of Calcium Ion in Ventricular Myocytes**

First the micro-cantilever and ventricular myocytes were adjusted to the same focal plane under the field of 10X objective lens, the ventricular myocytes with intact cell membrane, clear cytoplasm stripes and no vacuoles in the cell were selected to be placed in the center of the field of view. After the microcantilever raised by 100μm and the 40 oil mirror replaced, the micro-cantilever and cells were again adjusted to the same focal plane, and the micro-cantilever was adjusted to the cell surface. Final ran MetaMorph software, selected exposure time 200 ms of Laser-488 nm/530 nm and shoot for 5 minutes.

**Determination of Heart Weight and Collection of Tissue Samples**

After thoracotomy for aortic arch constriction, Penicillin (100,000 units/day) was injected for 3 consecutive days and rats in each group were weighed separately two days. Then the hearts of rats were taken out through thoracotomy, the residual blood was washed with PBS buffer, dried with filter paper, and the whole heart and left ventricular weights were weighed on the electronic balance, where cardiac hypertrophy index was equal to cardiac weight/body weight (mg/g).In addition, the physiological saline was pre-cooled for 2 hours in advance, and the left ventricular tissue was excised in vivo to cut into small pieces of 0.3 cm^3^, frozen and sealed with liquid nitrogen, and then stored at -80℃.

**High-throughput Sequencing of lncRNA in Atrial Cells and Ventricular Myocytes**

The left ventricular tissues of three rats in each group were randomly selected for high-throughput sequencing of lncRNA. After extracting total RNA from Trizol (Invitrogen, USA), rRNA was fragmented by kit, reverse-transcribed into single-stranded cDNA and purified. Subsequently, the end-repair and the linker primer were added with PCR amplification and purification. Then a library with fragment size of 260 bp was screened out through quality inspection and the library was qualitatively examined by Agilent 2200 TapeStation, and finally sequenced on the machine. Used Flow Cytometry technology and Illumina platform instrument (Hiseq3000/Hiseq 2500, Illumina, USA) for on-board operation and run the Pair End standard sequencing program. After obtaining the original sequencing data, it is first necessary to filter the data, remove the linker sequence and process the low-quality reads, and carry out sequencing quality evaluation and ribosomal RNA removal to obtain high-quality data, and compare it with the reference genome to obtain BAM files^[1-2]^. The gene expression level was calculated and the differentially expressed genes between the samples were analyzed and the difference of lncRNAs between the groups was screened by Fold Change.

**Analysis of Differential Gene Expression Levels**

Gene differential expression analysis is an independent statistical hypothesis test for thousands of genes, and a differential test P-value is obtained then the p-value is corrected by the FDR method ^[3]^. The lower the P-value or q-value, the more significant the difference in gene expression. We used Cuffquant and Cuffnorm software for expression analysis and used Cuffdiff software to analyze differentially expressed genes between samples.

**Multi-class Dif Analysis**

The random-variance model F-test was used to ﬁlter the differentially expressed genes for both the control and experiment groups. The differentially expressed genes were identiﬁed according to the p-value threshold (P < 0.05) and |log 2 FC|>=1. Log 2 FC is the differential expression multiple and takes the base 2 logarithm that log 2 FC>=1 is the up-regulated differential gene, and log 2 FC<=-1 is the down-regulated differential gene.

**Series Test of Cluster (STC) Analysis**

The series test of cluster algorithm of gene expression dynamics was used to proﬁle the gene expression time series and to select the most probable set of clusters generating the observed time series ^[4]^. We performed hierarchical clustering analysis on the selected differentially expressed genes to cluster genes with the same or similar expression behavior. After the differential expression genes were identiﬁed in a logical sequence according to RVM corrective ANOVA, a set of unique model expression proﬁles was selected in accordance with the different signal density change proﬁles of genes under different conditions. Next, the raw expression values were converted into log2 ratios, and the expression model proﬁles were related to the actual or expected number of genes assigned to each model proﬁle.

**Gene Ontology (GO) Analysis**

The Gene Ontology (GO) database （http://geneontology.org/ ）is a database created by the Gene Ontology Consortium^[5]^ and GO Analysis is applied to determine the differently expressed genes belonging to the main function according to the Gene Ontology, which is the key functional classiﬁcation of NCBI. In general, Fisher’s exact test and χ ^2^ test are applied to classify the GO category, and the false discovery rate (FDR) is calculated to correct the P-value.

**Different Expression of lncRNAs between SHR and WKY Rats**

Compared with the expression changes of lncRNAs in SHR group and WKY group, 52 kinds of lncRNAs were down-regulated and 86 kinds of lncRNAs were up-regulated. The red dots indicated lncRNAs whose expression were up-regulated by more than 2.0 times, and the green parts were down-regulated by more than 2.0 times (the significant difference between Q value < 0.05 or P value < 0.05 was statistically significant) ^[6-7]^.GO analysis was carried out on the genes |log2fold_change|>1 and Q< 0.05 with different expression levels between the two groups, and the analysis results were finally obtained ^[8-9]^. Please refer to the top ten enrichment classification maps of GO analysis (as shown in Figure.S2), and genes with obvious differential expression related myocardial mechanics between the two groups of rats were obtained.

**
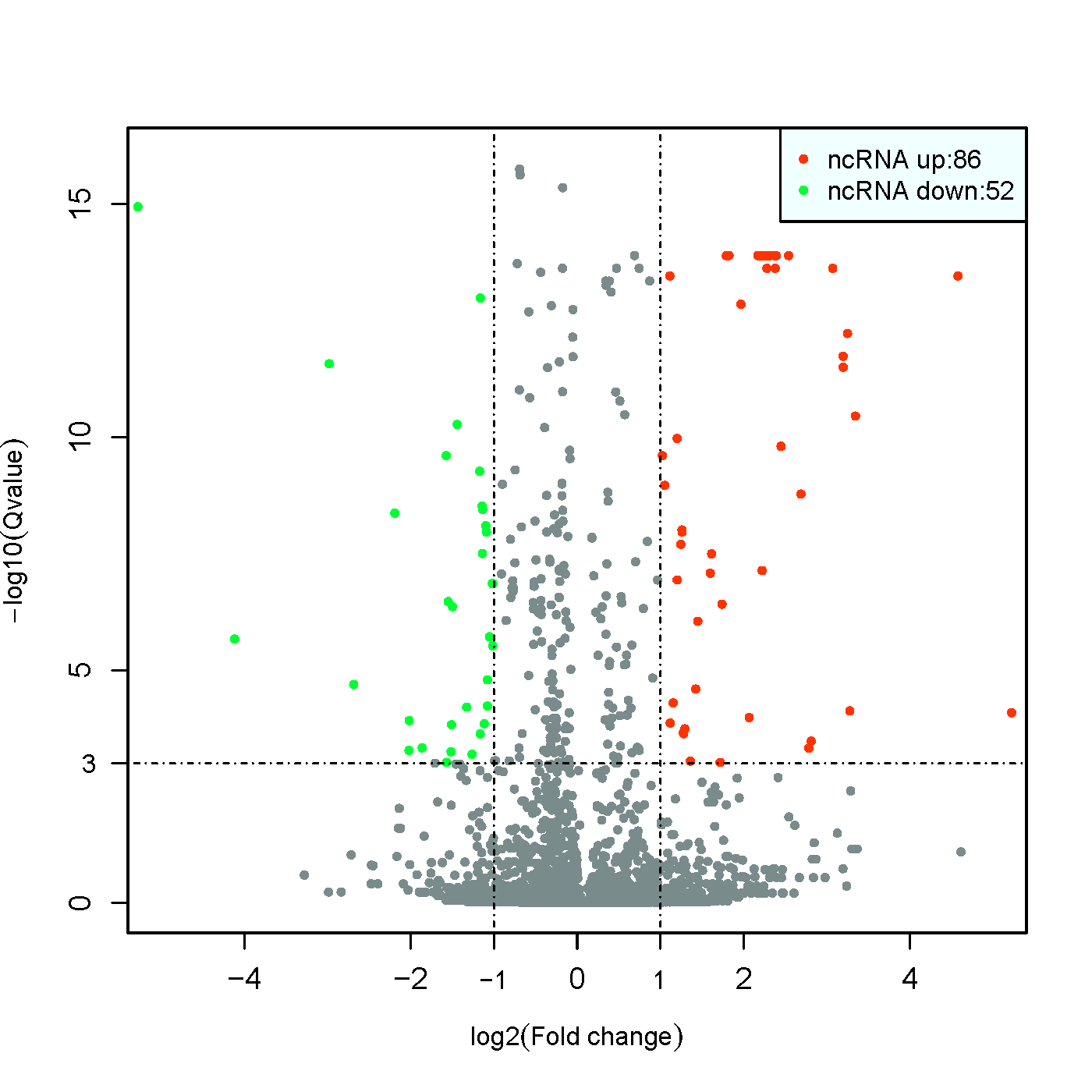
**

***Figure.S1*** Volcano plots of differentially expressed gene. The abscissa represented the multiple changes of gene expression in different samples and the ordinate represented the statistical significance of differentially expressed gene, which the red dot represented a significant up-regulation gene, and the green dot was a significant down-regulation gene, and the brown dots represented the genes that were not significantly different.


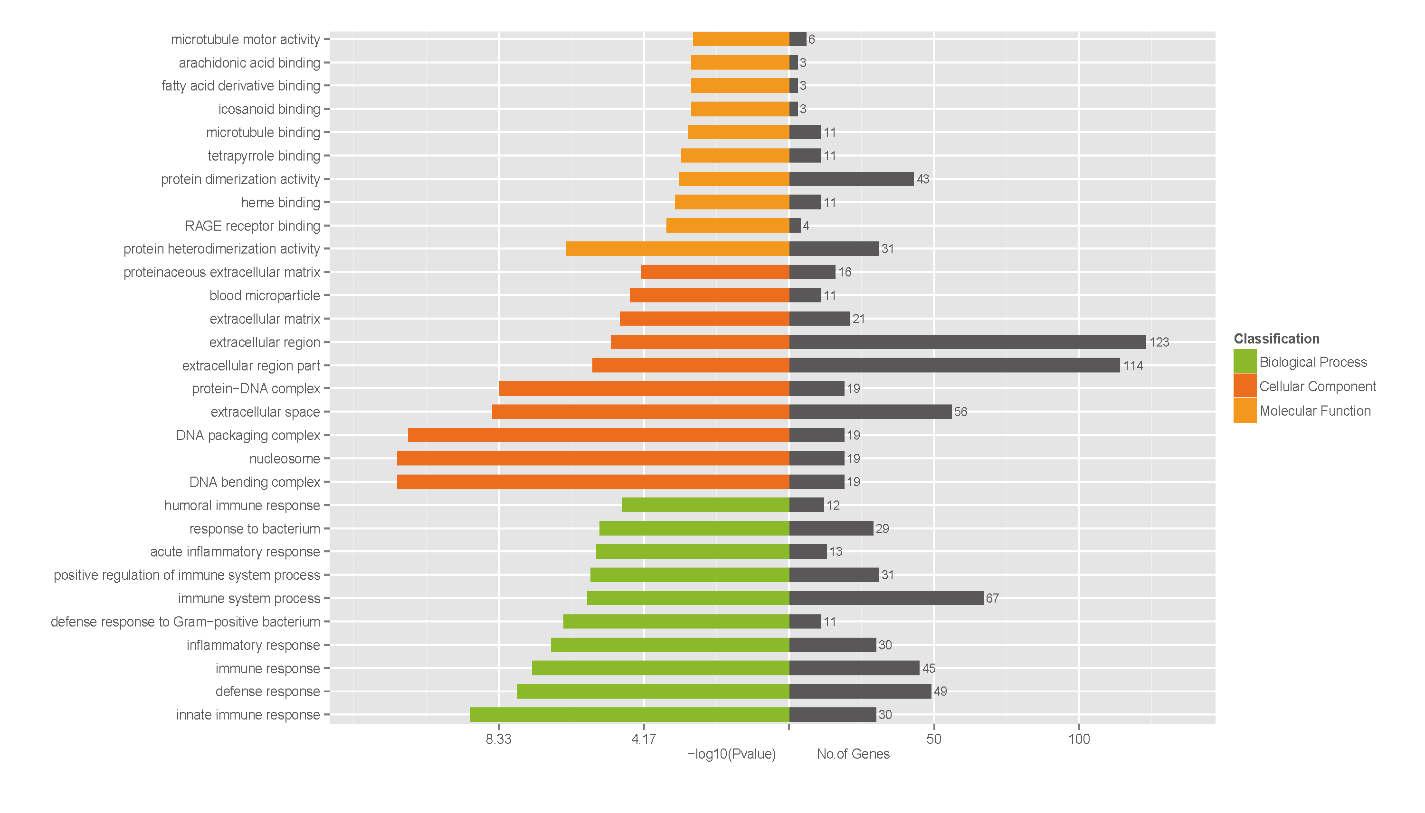
***Figure. S2*** GO analysis chart of lncRNAs comparison between SHR and WKY group rats (top ten items with most obvious differential expression). Green parts indicated genes related biological processes and the red represented cellular components, and the yellow represented molecular functions. In addition, the black represented the number of related genes.

**Supplementary Table S1**

|  | Fold Change | |  |
| --- | --- | --- | --- |
| lncRNA | WKY group | SHR group | p-value |
| XR_594989.1 | 0.005139103 | 0.538571333 | 0.000000 |
| XR_593234.1 | 0.0070609 | 0.264212667 | 0.000004 |
| XR_590412.1 | 0.29237 | 7.001696667 | 0.000000 |
| XR_360955.2 | 0.05649 | 0.575752667 | 0.000000 |
| XR_351096.2 | 0.064795133 | 0.629998667 | 0.000004 |
| XR_591693.1 | 0.0655894 | 0.625814 | 0.000000 |
| XR_592784.1 | 0.172140167 | 1.584363333 | 0.000000 |
| XR_591695.1 | 0.069348467 | 0.637704333 | 0.000000 |
| XR_354768.2 | 0.941679333 | 7.93776 | 0.000000 |
| XR_354090.2 | 0.047822933 | 0.336961667 | 0.000018 |
| XR_593235.1 | 0.052607233 | 0.363336333 | 0.000026 |
| XR_593690.1 | 0.236014667 | 1.52723 | 0.000000 |
| XR_597790.1 | 0.181753 | 1.061514333 | 0.000000 |
| XR_595802.1 | 0.231102333 | 1.267236667 | 0.000000 |
| XR_597773.1 | 0.177210333 | 0.933459333 | 0.000000 |
| XR_597792.1 | 0.151139667 | 0.78834 | 0.000000 |
| XR_597772.1 | 0.173490667 | 0.897934 | 0.000000 |
| XR_597782.1 | 0.160288 | 0.803116667 | 0.000000 |
| XR_597765.1 | 0.173021 | 0.858185667 | 0.000000 |
| XR_597781.1 | 0.156285333 | 0.775030667 | 0.000000 |
| XR_597785.1 | 0.162144333 | 0.80408 | 0.000000 |
| XR_597769.1 | 0.178541 | 0.880616 | 0.000000 |
| XR_597760.1 | 0.175239667 | 0.862183333 | 0.000000 |
| XR_597780.1 | 0.155226 | 0.761558 | 0.000000 |
| XR_597787.1 | 0.167265667 | 0.820482 | 0.000000 |
| XR_597784.1 | 0.161222 | 0.790835667 | 0.000000 |
| XR_597766.1 | 0.174138667 | 0.851787667 | 0.000000 |
| XR_597793.1 | 0.162847333 | 0.793607667 | 0.000000 |
| XR_597761.1 | 0.179176667 | 0.866805333 | 0.000000 |
| XR_597795.1 | 0.186087333 | 0.884225667 | 0.000000 |
| XR_597776.1 | 0.180578 | 0.852471 | 0.000000 |
| XR_597756.1 | 0.179564667 | 0.847505333 | 0.000000 |
| XR_597783.1 | 0.181159 | 0.853105333 | 0.000000 |
| XR_597788.1 | 0.186338667 | 0.877347667 | 0.000000 |
| XR_597774.1 | 0.183813333 | 0.864986667 | 0.000000 |
| XR_597771.1 | 0.181313667 | 0.853079667 | 0.000000 |
| XR_597767.1 | 0.179366667 | 0.843918667 | 0.000000 |
| XR_597764.1 | 0.185266667 | 0.871678 | 0.000000 |
| XR_597757.1 | 0.180268 | 0.848158333 | 0.000000 |
| XR_597755.1 | 0.179966667 | 0.846740333 | 0.000000 |
| XR_597762.1 | 0.172408 | 0.811176667 | 0.000000 |
| XR_597791.1 | 0.186993667 | 0.878255667 | 0.000000 |
| XR_597768.1 | 0.181322 | 0.848889333 | 0.000000 |
| XR_355734.1 | 0.380733333 | 1.781996667 | 0.000000 |
| XR_597759.1 | 0.179134 | 0.838176333 | 0.000000 |
| XR_597758.1 | 0.178838 | 0.836791333 | 0.000000 |
| XR_597777.1 | 0.194493667 | 0.895607667 | 0.000000 |
| XR_597786.1 | 0.176824 | 0.813692333 | 0.000000 |
| XR_597778.1 | 0.178287667 | 0.813331 | 0.000000 |
| XR_597794.1 | 0.177784333 | 0.803437 | 0.000000 |
| XR_597775.1 | 0.155786 | 0.703896667 | 0.000000 |
| XR_595452.1 | 1.395346667 | 5.98222 | 0.000000 |
| XR_358864.2 | 1.485733333 | 6.339783333 | 0.000000 |
| XR_360997.2 | 0.650050667 | 2.733393333 | 0.000005 |
| XR_595451.1 | 1.54343 | 6.47852 | 0.000000 |
| XR_352200.1 | 0.917598333 | 3.69096 | 0.000000 |
| XR_597779.1 | 0.148393667 | 0.582391 | 0.000000 |
| XR_597770.1 | 0.259302667 | 0.921646667 | 0.000000 |
| XR_597789.1 | 0.274343 | 0.974524333 | 0.000000 |
| XR_597763.1 | 0.253342667 | 0.880316 | 0.000000 |
| XR_595502.1 | 1.34059 | 4.580413333 | 0.000000 |
| XR_354997.2 | 0.1529318 | 0.512664667 | 0.000000 |
| XR_593341.1 | 0.0706509 | 0.233075333 | 0.000058 |
| XR_593342.1 | 0.073595567 | 0.242789667 | 0.000058 |
| XR_350321.2 | 0.197959333 | 0.608558667 | 0.000000 |
| XR_590162.1 | 0.204366667 | 0.621195 | 0.000000 |
| XR_352538.2 | 0.1954528 | 0.535883667 | 0.000000 |
| XR_596609.1 | 0.147707333 | 0.397831667 | 0.000001 |
| XR_595199.1 | 0.120357 | 0.310011 | 0.000054 |
| XR_595198.1 | 0.120449 | 0.310247 | 0.000054 |
| XR_356450.2 | 0.294763 | 0.725054 | 0.000010 |
| XR_596660.1 | 1.006045 | 2.44754 | 0.000012 |
| XR_596661.1 | 1.329910667 | 3.235446667 | 0.000012 |
| XR_362531.2 | 0.185175 | 0.449868 | 0.000011 |
| XR_591249.1 | 0.480591 | 1.155787333 | 0.000000 |
| XR_592022.1 | 0.269324667 | 0.646547667 | 0.000000 |
| XR_591250.1 | 0.514026667 | 1.222792 | 0.000000 |
| XR_356917.2 | 0.634104 | 1.462936667 | 0.000000 |
| XR_595188.1 | 0.515852333 | 1.190047667 | 0.000000 |
| XR_592268.1 | 1.046910333 | 2.336426667 | 0.000002 |
| XR_590677.1 | 0.964519667 | 2.140976667 | 0.000000 |
| XR_592270.1 | 1.167916667 | 2.543693333 | 0.000007 |
| XR_592269.1 | 1.16356 | 2.534203333 | 0.000007 |
| XR_595117.1 | 1.005618333 | 2.182913333 | 0.000000 |
| XR_592911.1 | 1.380368 | 2.87119 | 0.000000 |
| XR_358018.2 | 1.542673333 | 3.14776 | 0.000000 |
| XR_596712.1 | 0.930029667 | 0.462301667 | 0.000000 |
| XR_595803.1 | 2.772633333 | 1.375343333 | 0.000000 |
| XR_596711.1 | 0.818726333 | 0.405041 | 0.000000 |
| XR_146205.3 | 13.90826667 | 6.844156667 | 0.000000 |
| XR_597042.1 | 0.911493333 | 0.440731333 | 0.000000 |
| XR_592136.1 | 1.003768 | 0.476813667 | 0.000001 |
| XR_592300.1 | 0.373529667 | 0.177398333 | 0.000003 |
| XR_353278.2 | 0.670019 | 0.315493667 | 0.000000 |
| XR_592102.1 | 0.668968 | 0.314998667 | 0.000000 |
| XR_592101.1 | 0.701677333 | 0.330400333 | 0.000000 |
| XR_592100.1 | 0.620214333 | 0.290338 | 0.000000 |
| XR_592098.1 | 0.620855667 | 0.290637667 | 0.000000 |
| XR_592060.1 | 0.229217333 | 0.106049967 | 0.000007 |
| XR_592099.1 | 0.773161667 | 0.352772333 | 0.000000 |
| XR_589908.1 | 0.653243667 | 0.297539333 | 0.000000 |
| XR_592103.1 | 1.088949 | 0.494589 | 0.000000 |
| XR_146216.3 | 2.65996 | 1.19083 | 0.000000 |
| XR_592061.1 | 0.239514 | 0.107053967 | 0.000013 |
| XR_349933.2 | 0.656405333 | 0.292225 | 0.000000 |
| XR_355819.2 | 79.65666667 | 34.3878 | 0.000000 |
| XR_355820.2 | 81.39156667 | 35.06833333 | 0.000000 |
| XR_146107.3 | 1.866706667 | 0.780233667 | 0.000037 |
| XR_596798.1 | 0.474594 | 0.189486 | 0.000003 |
| XR_597299.1 | 1.113434667 | 0.423227333 | 0.000000 |
| XR_596966.1 | 0.410650667 | 0.151524333 | 0.000000 |
| XR_591718.1 | 29.2579 | 10.6365 | 0.000000 |
| XR_593737.1 | 1.409266667 | 0.499947 | 0.000000 |
| XR_360864.1 | 0.448276333 | 0.157870333 | 0.000008 |
| XR_353596.2 | 0.168796367 | 0.059204433 | 0.000032 |
| XR_362157.2 | 2.740963333 | 0.938854333 | 0.000000 |
| XR_146396.2 | 0.276305 | 0.093486833 | 0.000058 |
| XR_597014.1 | 0.285070333 | 0.0964524 | 0.000058 |
| XR_591618.1 | 1.137470667 | 0.383178833 | 0.000000 |
| XR_597429.1 | 1.055661333 | 0.319497333 | 0.000000 |
| XR_597427.1 | 1.039686 | 0.313828 | 0.000000 |
| XR_597340.1 | 20.3815 | 6.131956667 | 0.000000 |
| XR_597013.1 | 0.291443333 | 0.0803776 | 0.000026 |
| XR_594204.1 | 0.439771 | 0.1087922 | 0.000006 |
| XR_350590.1 | 0.399388333 | 0.0986654 | 0.000030 |
| XR_362539.1 | 1.033707 | 0.226988 | 0.000000 |
| XR_597428.1 | 1.001106333 | 0.208897 | 0.000000 |
| XR_597430.1 | 1.045759 | 0.216381333 | 0.000000 |
| XR_146082.2 | 1.866506667 | 0.359428667 | 0.000000 |
| XR_595339.1 | 0.83153 | 0.129558 | 0.000001 |
| XR_591652.1 | 0.629981667 | 0.0800272 | 0.000000 |
| XR_597343.1 | 0.442746 | 0.0255979 | 0.000000 |
| XR_353580.2 | 1.711996667 | 0.068522533 | 0.000000 |
| XR_593654.1 | 2.21731 | 0.064291733 | 0.000000 |
| XR_590077.1 | 0.479053 | 0.012361867 | 0.000000 |
| XR_006462.6 | 0.561277 | 0.01156296 | 0.000000 |
| XR_591450.1 | 63.43593333 | 0.414745333 | 0.000000 |
| XR_590849.1 | 3.753423333 | 0.009439133 | 0.000000 |

***Table.S1*** List of lncRNAs Differentially Expressed between SHR and WKY Rats.

86 LncRNAs are up-regulated in red color and 52 LncRNAs are down-regulated in green color.

**Supplementary Table S2**

| Biological Processes Related to Cardiac Mechanics | Number | P value | Name of participating gene |
| --- | --- | --- | --- |
| Regulation of apoptosis in striated muscle cells | 2 | 0.047 | Edn1;Nrg1 |
| Regulation of muscle compliance | 2 | 0.047 | Edn1;Bmp10 |
| Regulation of cardiac growth | 2 | 0.045 | Bmp10;Nrg1 |
| Ventricular muscle tissue development | 2 | 0.039 | Bmp10;Nrg1 |
| Myocardial cell proliferation | 2 | 0.039 | Bmp10;Nrg1 |
| Myocardial tissue development | 4 | 0.037 | Bmp10;Ankrd1;Nrg1;Tcap |
| Histomorphology of ventricular muscle | 2 | 0.033 | Bmp10;Nrg1 |
| Differentiation of cardiomyocytes | 3 | 0.030 | Bmp10;Nrg1;Tcap |
| Integrin-mediated Regulation of Cell Adhesion | 2 | 0.029 | Cxcl13;Serpine1 |
| Rhabdomyocyte development | 4 | 0.028 | Edn1;Bmp10;Krt19;Tcap |
| Regulation of myocardial cell proliferation | 2 | 0.026 | Bmp10;Nrg1 |
| Negative Regulation of Cardiomyocyte Apoptosis | 2 | 0.026 | Edn1;Nrg1 |
| Muscle system process | 6 | 0.017 | Bmp10;Scn3b;Edn1;Sln;Ctgf;Tcap |
| Regulation of striated muscle contraction | 3 | 0.017 | Ctgf;Bmp10;Scn3b |
| Calcium ion reaction | 4 | 0.014 | Alox15;Fos;Txnip;Edn1 |
| Aging | 7 | 0.013 | Adra1d;Edn1;Fos;Ctgf;  Hcn2;Cyp1a1;Serpine1 |
| Positive regulation of heart rate | 2 | 0.013 | Edn1;Scn3b |
| Positive Regulation of Myocardial Cell Proliferation | 2 | 0.013 | Bmp10;Nrg1 |
| Regulation of myocardial contraction | 3 | 0.011 | Ctgf;Bmp10;Scn3b |
| Ventricular cardiomyocyte differentiation | 2 | 0.009 | Bmp10;Nrg1 |
| Myocardial tissue contraction | 4 | 0.003 | Ctgf;Bmp10;Scn3b;Tcap |
| Blood pressure regulation | 6 | 0.002 | Nrg1;Hba2;Edn1;Apln;  Adra1d;Hba1 |
| Regulation of sarcomere structure | 2 | 0.002 | Edn1;Bmp10 |
| Muscle morphology | 4 | 0.001 | Bmp10;Ankrd1;Nrg1;Tcap |
| Positive regulation of myocardial contractility | 3 | 0.001 | Edn1;Hcn2;Scn3b |
| Negative regulation of blood pressure | 4 | 0.001 | Hba2;Adra1d;Hba1;Apln |
| Muscle histomorphology | 4 | 0.001 | Bmp10;Ankrd1;Nrg1;Tcap |
| Structural composition of actin | 4 | 0.001 | Edn1;Bmp10;Krt19;Tcap |
| Positive regulation of blood circulation | 5 | 0.001 | Adra1d;Edn1;Hcn2;Apln;Scn3b |
| Myocardial histomorphology | 4 | 0.001 | Bmp10;Ankrd1;Nrg1;Tcap |
| System process control | 11 | 0.000 | Adra1d;Bmp10;Edn1;Scn3b;Hba2;Hcn4;Apln;Sln;Ctgf;Hcn2;Hba1 |
| Myofibril aggregation | 4 | 0.000 | Edn1;Bmp10;Krt19;Tcap |
| Regulation of blood circulation | 8 | 0.000 | Adra1d;Bmp10;Edn1;Scn3b;Hcn4;Apln;Ctgf;Hcn2 |
| Cardiac contraction regulation | 8 | 0.000 | Adra1d;Bmp10;Edn1;Scn3b;Hcn4;Apln;Ctgf;Hcn2 |
| Cyclic system process | 13 | 0.000 | Angpt1;Bmp10;Edn1;Adra1d;Nrg1;Scn3b;Hba2;Hcn4;Apln;Ctgf;Hcn2;Hba1;Tcap |
| Blood pressure circulation | 13 | 0.000 | Angpt1;Bmp10;Edn1;Adra1d;Nrg1;Scn3b;Hba2;Hcn4;Apln;Ctgf;Hcn2;Hba1;Tcap |
| Heart cycle | 9 | 0.000 | Adra1d;Bmp10;Edn1;Scn3b;  Hcn4;Apln;Ctgf;Hcn2;Tcap |
| systole | 9 | 0.000 | Adra1d;Bmp10;Edn1;Scn3b;Hcn4;Apln;Ctgf;Hcn2;Tcap |
| Drug response | 18 | 0.000 | Edn1;Txnip;Lcn2;Atp1a3;Mmp12;Cyp1a1;Cyp2e1;Bdh1;Tgif1;Fos;Card9;Alas2;Tnfrsf11b;Serpine1;Ankrd1;Wfdc1;Hcn2;Mt2A |

***Table.S2*** Expression Differences of Cardiac Mechanics Related Genes in SHR Group vs. WKY Group.

**Supplementary Table S3**

|  | Fold Change | |  |
| --- | --- | --- | --- |
| lncRNA | WKY group | WKY- Amiodarone | p-value |
| XR_362539.1 | 1.033707 | 0.308323333 | 0.000002 |
| XR_355816.2 | 0.771765333 | 0.25427 | 0.000001 |
| XR_592099.1 | 0.773161667 | 0.265640333 | 0.000000 |
| XR_592100.1 | 0.620214333 | 0.213600333 | 0.000000 |
| XR_592098.1 | 0.620855667 | 0.213821333 | 0.000000 |
| XR_592103.1 | 1.088949 | 0.37547 | 0.000000 |
| XR_592102.1 | 0.668968 | 0.231743333 | 0.000000 |
| XR_592101.1 | 0.701677333 | 0.243074667 | 0.000000 |
| XR_353278.2 | 0.670019 | 0.232107667 | 0.000000 |
| XR_597430.1 | 1.045759 | 0.382415 | 0.000000 |
| XR_597428.1 | 1.001106333 | 0.366757667 | 0.000000 |
| XR_595803.1 | 2.772633333 | 1.257677333 | 0.000004 |
| NR_073147.1 | 2.474093333 | 1.146588333 | 0.000000 |
| XR_592737.1 | 0.747554 | 0.362830333 | 0.000009 |
| XR_592738.1 | 0.772403333 | 0.375645667 | 0.000012 |
| XR_354337.1 | 0.783603667 | 0.386889667 | 0.000025 |
| XR_354338.1 | 0.787902 | 0.390129333 | 0.000034 |
| XR_591618.1 | 1.137470667 | 2.31769 | 0.000000 |
| XR_595813.1 | 0.193299333 | 0.395895667 | 0.000001 |
| XR_597143.1 | 0.215122667 | 0.449948 | 0.000003 |
| XR_595814.1 | 0.201688 | 0.426663 | 0.000001 |
| XR_592651.1 | 9.713026667 | 22.5512 | 0.000001 |
| NR_027324.1 | 3.80987 | 10.21648667 | 0.000000 |
| XR_596501.1 | 0.093046933 | 0.2670401 | 0.000005 |
| XR_593513.1 | 0.309644667 | 1.021769 | 0.000000 |
| XR_592022.1 | 0.269324667 | 0.993578667 | 0.000000 |

***Table.S3*** List of lncRNA Differentially Expressed before and after Amiodarone Treatment in Arrhythmic WKY Rats. Nine LncRNAs are up-regulated in red color and seventeen LncRNAs are down-regulated in green color.

**Supplementary Table S4**

|  | Fold Change | |  |
| --- | --- | --- | --- |
| lncRNA | SHR group | SHR-Amiodarone | p-value |
| XR_591602.1 | 22.41093333 | 11.14606 | 0.000000 |
| XR_358909.2 | 0.311299 | 0.649763333 | 0.000011 |
| XR_358910.2 | 0.310863 | 0.648854333 | 0.000011 |
| XR_590319.1 | 0.156503667 | 0.336688 | 0.000031 |
| XR_597308.1 | 0.514704 | 1.16896 | 0.000012 |
| XR_592190.1 | 1.958516667 | 7.279866667 | 0.000000 |

***Table.S4*** List of lncRNAs Differentially Expressed with Amiodarone Treatment in Arrhythmic SHR Rats. Five LncRNAs are up-regulated in red color and only one is down-regulated in green color.

**Supplementary Figure S3**


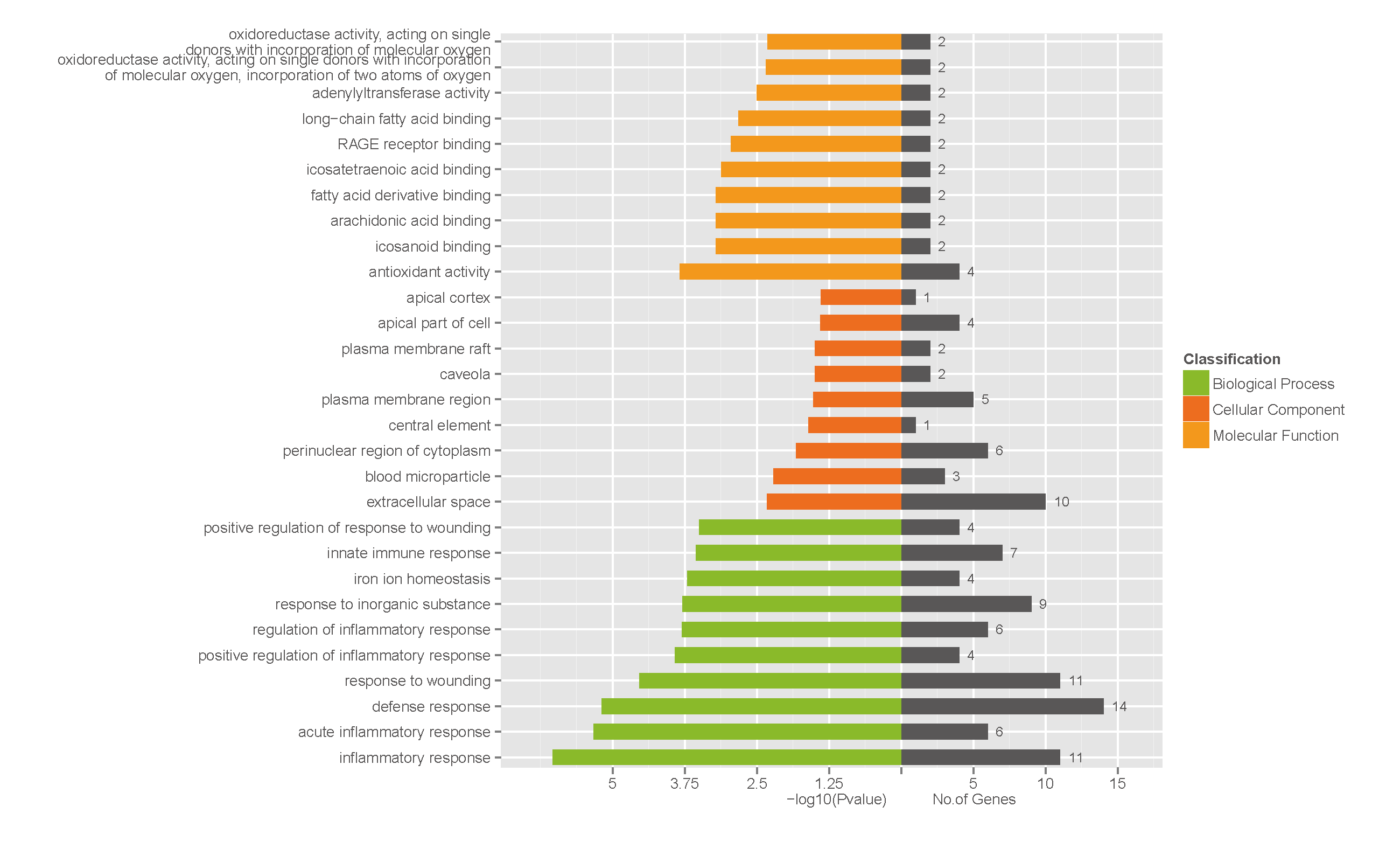


***Fig.S3*** GO analysis chart of lncRNAs comparison before and after amiodarone treatment in WKY group (top ten items with most obvious differential expression). Green parts indicated genes related biological processes and the red represented cellular components, and the yellow represented molecular functions. In addition, the black represented the number of related genes.

**References:**

1. Batista PJ, Chang HY. Long Noncoding RNAs: Cellular Address Codes in Development and Disease. ***Cell,*** 152(6): 1298-1307(2013).
2. Helgadottir A, Thorleifsson G, Manolescu A. A common variant on chromosome 9p21 affects the risk of myocardial infarction. ***Science***,316(5830): 1491-1493(2007).
3. Reuter JA, Spacek DV, Pai RK, et al. Simul-seq: combined DNA and RNA sequencing for whole-genome and transcriptome profiling. ***Nature Methods****,*13, 953-958(2016).
4. Rui C, Mias G I, Jennifer LPT, et al. Personal Omics Profiling Reveals Dynamic Molecular and Medical Phenotypes. ***Cell***, 148(6):1293-1307(2012).
5. Carbon S, Chan J, Kishore R, et al. Expansion of the Gene Ontology knowledgebase and resources. ***Nucleic Acids Research****(*2017).
6. Klattenhoff CA, Scheuermann JC, Surface LE, et al. Braveheart, a long noncoding RNA required for cardiovascular lineage commitment. ***Cell,***152(3): 570(2013).
7. Kung JT, Colognori D, Lee JT. Long noncoding RNAs: past, present, and future. ***Genetics,***193(3): 651(2013).
8. Helgadottir A, Thorleifsson G, Manolescu A, et al. A common variant on chromosome 9p21 affects the risk of myocardial infarction.***Science,*** 316(5830): 1491-1493(2003).
9. Batista PJ, Chang HY. Long Noncoding RNAs: Cellular Address Codes in Development and Disease. ***Cell***, 152(6): 1298-1307(2013).
